# Impact of Probiotic Intervention on Lymphocyte Activity in Mice Challenged with Pea Albumins (*Pisum sativum* L.)

**DOI:** 10.3390/nu18203403

**Published:** 2026-10-17

**Authors:** Justyna Chudzik-Kozłowska, Ewa Wasilewska, Anita Mikołajczyk, Dagmara Złotkowska

**Affiliations:** 1 InLife Institute of Animal Reproduction and Food Research, Polish Academy of Sciences, 10-683 Olsztyn, Poland; 2 Department of Psychology and Sociology of Health and Public Health, Collegium Medicum, University of Warmia and Mazury in Olsztyn, 10-082 Olsztyn, Poland; a.mikolajczyk@uwm.edu.pl

**Keywords:** probiotic, pea albumins, T cells, mucosal immune system

## Abstract

**Background:** Probiotics are microorganisms with proven health benefits in humans. However, the mechanisms underlying the direct effects of these compounds on immune cells remain unclear. This study aimed to examine the effects of probiotics on immunity in mice exposed to allergenic dietary proteins, specifically pea albumin (PA). We hypothesized that probiotics would alter lymphocyte activity, leading to measurable differences in the immune response to these proteins. **Methods:** The relationship between probiotic intervention and the adaptive immune response to PA was tested in Balb/c and C57BL/6 mice to account for strain differences. ELISA and flow cytometry were used to determine specific immunoglobulin titers, lymphocyte profile, including CD4^+^/CD8^+^ ratio, and cytokine release. **Results:** We observed higher levels of pea albumin-specific immunoglobulins in the serum and fecal extracts of Balb/c mice than in those of C57BL/6 mice. Probiotic intervention significantly impacted serum and fecal IgA levels (*p* < 0.05). We found that probiotic intervention generally increased the percentage of CD4^+^, CD4^+^ CD25^+^, and CD8^+^ T cells in the Peyer’s patches, mesenteric lymph nodes, spleen, and head and neck lymph nodes of Balb/c mice, which was not observed in C57BL/6 mice. In ex vivo cultures, lymphocytes from probiotic-treated Balb/c mice responded to PA stimulation by significantly altering the levels of secreted cytokines (*p* < 0.05). The Balb/c and C57BL/6 mice, administrated probiotic preparation and PA, presented differences in the SCFA profiles (*p* < 0.05). **Conclusions:** the multivalent probiotic preparation significantly modulated humoral and cellular immune responses against PA in Balb/c mice. This suggests that it may be an important tool for the targeted modulation of the immune response to dietary proteins.

## 1. Introduction

The global prevalence of food allergies is increasing, as reflected in cow’s milk allergy (CMA) statistics. The self-reported prevalence of CMA ranges from 1.2% to 17%, depending on age, whereas confirmed IgE sensitization is estimated to be 2–9% [1]. This increase has driven many patients and consumers to avoid cow milk proteins and seek plant-based alternatives, such as pea proteins. However, replacing milk with plant-based products exposes consumers to potentially allergenic proteins, such as peanut proteins. In European countries, 3.3% of the allergic population is allergic to beans, and 0.8% is allergic to green peas (*Pisum sativum* L.). Although pea allergy is less common than allergy to other legumes, such as peanuts or soybeans, cross-reactivity among legume proteins can broaden sensitization and exacerbate clinical symptoms. Nevertheless, interest in alternative protein sources continues to grow, along with the likelihood of triggering allergies to proteins, such as those from peas [2].

The pea albumin fractions responsible for pea allergy are Pis s1 (44 kDa), Pis s2 (63 kDa), and Pis s3 (9.5 kDa), which are listed in the WHO/IUIS Allergen Nomenclature database (https://allergen.org, accessed on 2 April 2026). Our previous studies demonstrated that pea albumins (PAs) isolated from *Pisum sativum* L., including Pis s1 and Pis s2, induced immune responses in Balb/c and C57BL/6 mice [3]. A 10-day oral administration of PA increased anti-PA IgG titers in both strains, whereas specific IgA was mainly induced in Balb/c mice, with C57BL/6 mice showing only very low sIgA levels. At the cellular level, we observed an increased number of CD4^+^ lymphocytes in the mesenteric lymph nodes (MLNs) and Peyer’s patches (PPs), which are key inductive sites for immune responses to food allergens, in both mouse strains [3]. The observed antigenic potential of PA highlights the need to better understand their immunogenic mechanisms, particularly to enable the responsible design of personalized foods for individuals at risk of food allergies [4].

Gut-associated lymphoid tissue (GALT), comprising the lamina propria, MLNs, PPs, and isolated lymphoid follicles, is central to the immune system’s “dialogue” with incoming food components and intestinal microbiota. Continuous exposure to dietary antigens and commensal or ingested bacteria forces GALT to maintain intestinal homeostasis by balancing efficient nutrient uptake with protection against harmful agents. Intestinal homeostasis is supported by several structures that differ in their accessibility to bacteria, metabolites, and dietary antigens. The first intestinal defense is the mucus layer and secretory IgA (sIgA), which are crucial for immune exclusion and shaped by tolerogenic signals from goblet cells and CD103^+^ dendritic cells in the lamina propria [5]. The intestinal epithelial barrier, including the subepithelial lamina propria, which is rich in immune cells such as B and T (CD4^+^ and CD8^+^) lymphocytes, eosinophils, dendritic cells (DCs), mast cells, macrophages, and connective tissue components, is the main site of adaptive immunity. The gut vascular barrier (GVB) restricts the passage of molecules larger than approximately 4 kDa [6]. The GVB also enables DC migration to MLNs to support tolerogenic responses and regulates leukocyte trafficking by upregulating endothelial adhesion molecules and activating circulating immune cells, thereby engaging the gut-associated immune response [7]. In addition, the gut microbiota is a key modulator of the gut–brain–immune axis. Microbial communities influence intestinal homeostasis through metabolites and bacterial products, such as short-chain fatty acids (SCFAs), small peptides, and neurotransmitter-binding molecules, including dopamine and serotonin [8]. Together, these components constitute crucial elements of the communication pathways along the gut–brain axis [9].

Among immune cells, CD4^+^ T cells are central to adaptive immunity and coordinate effector responses through cytokine secretion and interactions with other immune cells. CD4^+^ T cells recognize antigens presented by antigen-presenting cells (APCs) via major histocompatibility complex class II (MHC-II) molecules. Oral tolerance, a state in which oral exposure to antigens leads to suppression rather than activation of immune responses, relies heavily on CD4^+^ T cells, particularly regulatory T cells (Tregs), which suppress effector T cell responses and maintain immune tolerance to food antigens [10].

Probiotics are microorganisms that support gut health and modulate immune responses when administered in sufficient amounts. Numerous studies have indicated that probiotics interact with CD4^+^ T cells and influence their functions [11]. Probiotic intervention activates regulatory cells and exerts beneficial health effects [12], whereas another study reported that probiotics can attenuate the pathogenic effects of lipopolysaccharide (LPS) on immune cell responses [13]. These observations are consistent with an earlier study, which demonstrated that yogurt starter strains can modulate the antigenicity of milk proteins [14]. Collectively, these data suggest that probiotic interventions can modulate immune responses to orally administered antigens. Probiotics secrete a variety of molecules into the intestinal milieu, including surface-associated and secreted proteins, peptides, amino acids, and SCFAs [12]. These mediators contribute to the complex crosstalk between the gut microbiome, host immune system, and epithelial cells [12].

Probiotics influence the functioning of the immune system by directly interacting with intestinal epithelial cells and immune cells, strengthening the intestinal barrier, and modulating both innate and adaptive immune responses [12]. Pea protein is a nutritionally valuable source of highly bioavailable protein, rich in essential amino acids [15,16]. However, its protein fractions can act as allergens or neoallergens formed during food processing. Because probiotics can strengthen the intestinal barrier and modulate immune response, they may influence how the immune system reacts to pea proteins. This makes their combined use relevant for nutrient absorption, intestinal barrier integrity, and prevention of food allergies.

Despite these findings, the antigenicity of pea proteins, not albumin alone, and the immunomodulatory properties of probiotics have so far been investigated independently [12,17]. It remains unclear whether probiotic administration can modify cellular and humoral immune responses directed against pea albumins, and whether any such effect depends on the host’s immunological background. Regimen-related questions, such as probiotic dose and the timing of supplementation relative to antigen exposure, remain open and were not addressed here. This study tested a single multi-strain preparation administered before and together with the antigen.

Balb/c and C57BL/6 mice differ in the genetic basis of their immune pathways: Balb/c mice show a predominance of Th2-mediated immunity, whereas C57BL/6 mice show a predominance of Th1-mediated immunity [18,19,20]. Our earlier study showed that both strains responded differently to pea albumins [3]. Therefore, we deliberately chose these two immunologically contrasting strains to test whether the effect of probiotic supplementation on the response to PA depends on the host immunological background.

This study aimed to determine how probiotic administration affects the development of humoral and cellular immune responses to orally administered PA in Balb/c and C57BL/6 mice and to test the hypothesis that probiotic intervention differentially modulates PA-specific immunity in these two strains.

## 2. Materials and Methods

### 2.1. Pea Albumins Preparation

Pea (*Pisum sativum* L.) seeds were obtained from the Plant Breeding Station Łagiewniki, Poland. Pea albumins (PAs) were isolated and characterized as previously described [3]. Briefly, pea flour was dissolved in acetate buffer (0.1 M; pH 4.9) and stirred for 24 h at 4 °C. The mixture was then centrifuged, dialyzed against water, and lyophilized. The final purification of the PA fraction was performed using DEAE ion-exchange chromatography, confirming the presence of the main pea allergens, Pis s1 and Pis s2. The PA lyophilizate was divided into small portions, packed in airtight doypack-type bags, and stored in a refrigerator for further use and analysis.

### 2.2. Probiotic Preparations

Two commercial probiotic preparations were used: PP1 contained *Lactococcus lactis* LI-23, *Lactobacillus plantarum* Lp-115, *Lactobacillus rhamnosus* GG, *Streptococcus thermophilus* St-21, *Bifidobacterium breve* Bb-03, *Lactobacillus casei* Lc-11, *Bifidobacterium bifidum* Bb-02, *Bifidobacterium animalis*, and *Lactobacillus acidophilus* LA-14, and PP2 contained *Lactobacillus rhamnosus* GG. Suspensions for oral administration were freshly prepared before use by dissolving the tablets in sterile phosphate-buffered saline (PBS; 0.1M, pH 7.2) to a final concentration of 2.4 × 10^8^ bacterial cells per 250 μL per mouse, based on the manufacturer’s declaration of viable cell counts.

### 2.3. Biological Experiments

Female Balb/c and C57BL/6 mice (6 weeks old) were obtained from the Institute of Experimental Medicine, Medical University of Warsaw (Warsaw, Poland). Mice were housed in individually ventilated cages (IVC system, M.A.C.S.^®^, Alternative Design Manufacturing and Supply Inc., Siloam Springs, AR, USA) with a 15-air changes per hour ventilation rate, in the animal facility of the Institute of Animal Reproduction and Food Research of PAS (Olsztyn, Poland) under standard conditions (12 h light/12 h dark cycle, 22–24 °C, 45–64% relative humidity). Separately, for each strain, mice of comparable age and body weight were placed in cages of 5, without a formal randomization procedure. The animals were fed standardized, cereal-based natural-ingredient diets (Altromin Spezialfutter GmbH & Co. KG, Lage, Germany) ad libitum. Breeding mice and young animals up to 8 weeks of age received Altromin 1314 TPF breeding diet (10 mm pellets, total pathogen free). Adult mice (from 8 weeks of age onward) were maintained on Altromin 1324 TPF maintenance diet (10 mm pellets, total pathogen free). Both diets are fixed-formula, natural-ingredient diets based on soy, wheat, and corn, and are explicitly free of alfalfa and fish/animal meal, as well as deficient in nitrosamines. The Altromin 1314 formulation provides ~22.5% crude protein, ~5.1% crude fat, ~4.5% crude fiber, and ~6.1% crude ash (metabolized energy: 27% protein, 14% fat, 59% carbohydrates). The Altromin 1324 formulation provides ~19.2% crude protein, ~4.1% crude fat, ~6.1% crude fiber, and ~5.9% crude ash (metabolized energy: 24% protein, 11% fat, 65% carbohydrates. The animals had ad libitum access to sterile drinking water throughout the experimental period. All procedures were approved by the Local Ethics Committee University of Warmia and Mazury in Olsztyn, Poland (permit no. 80/2011/N). All procedures were classified as moderate severity according to the approved protocol. No unexpected adverse events occurred during the study. No predefined humane endpoints requiring early euthanasia were established, as animals remained in good clinical condition throughout the experimental period and no signs requiring premature termination were observed. No animals were excluded from the experiment, and all animals were included in the final analyses.

**Figure 1:**
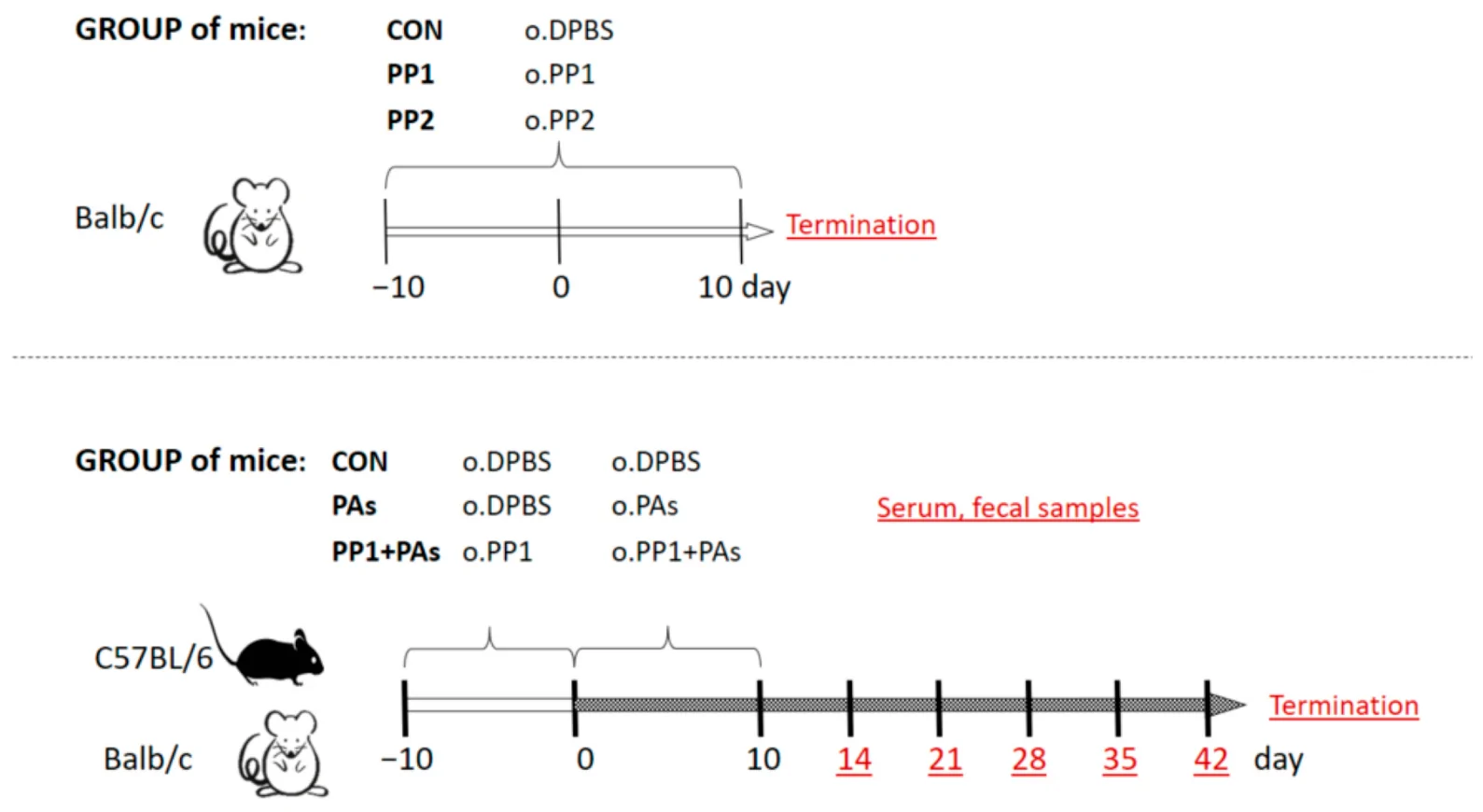
Schematic representation of the experimental procedure. Abbreviations: PA, pea albumins; PP1, PP2, probiotic preparations; PBS, phosphate-buffered saline; o., oral gavage.

Biological experiments were conducted in two steps (Figure 1). In the first step, a pre-experiment was performed to select the probiotic preparation: Balb/c mice (*n* = 5 per group) were orally administered PP1, PP2, or PBS (vehicle control) to assess the effects of probiotic intervention on the CD4^+^/CD8^+^ T cell ratio in healthy, non-sensitized animals. Balb/c mice were used for the pre-experiment because they showed a stronger response to PA than C57BL/6 mice in a previous study [3]. Therefore, we considered Balb/c mice as a more sensitive model for detecting the immunomodulatory effects of the candidate probiotic. The purpose of this screening step was to select a preparation with higher biological activity, not to compare the strains. In line with the 3R principle, screening was restricted to a single strain to limit animal numbers. The selected preparation was then evaluated in both strains. Based on the pre-experiment, we used PP1 for the main experiment in both Balb/c and C57BL/6 mice (Figure 1). The mice were then assigned to the following groups within each strain: PBS (control), PA (pea antigen only), and PP1 + PA (probiotic preparation 1 and pea antigen). For 10 consecutive days prior to antigen administration, mice in the PP1 + PA group received 250 μL of PP1 suspension per mouse per day via oral gavage, whereas those in the PA and PBS groups received the same volume of PBS per mouse. During the subsequent 10 days, mice in the PP1 + PA group received PP1 together with the tested antigen (200 μg PA/mouse per dose) by oral gavage; mice in the PA group received 200 μg PA/mouse in PBS, and the PBS mice continued to receive only PBS. Cholera toxin (CT, 5 μg/mouse) was added as a mucosal adjuvant to probiotic/antigen or antigen/PBS mixtures on days 1, 3, and 5 of antigen administration.

Blood and fecal samples were collected weekly from individual mice to monitor humoral responses. Mice were euthanized by isoflurane inhalation on the 42nd day of the experiment. Single-cell suspensions were prepared from the spleen (SPL), head and neck lymph nodes (HNLNs), mesenteric lymph nodes (MLNs), and Peyer’s patches (PPs) for further analysis (p.2.4). Each experiment was repeated twice (five mice per group in each repetition; *n* = 10 mice per group in total). Individual mice were used as experimental units for the humoral response analysis. Because the interventions (probiotic and antigen administration) were performed by investigators handling the animals, formal blinding was not applied during treatment.

### 2.4. Sample and Tissue Collection and Lymphocyte Isolation

Tissue collection and lymphocyte isolation were performed as previously described [3]. After euthanasia, the SPLs, HNLNs, MLNs, and PPs were aseptically excised. The tissues were homogenized in a Dounce tissue homogenizer, washed with cold phosphate-buffered saline (PBS; 0.1M, pH 7.4), and centrifuged. Splenocytes were additionally treated with red cell lysis buffer (555899; BD Bioscience, San Jose, CA, USA). The tissues from each experimental group were pooled (3, 3, and 4), resulting in three replicates for SPL, HNLN, MLN, and PP for flow cytometry analysis (FC) for each experimental group. The remaining cells (after subtracting 10^5^ cells designated for FC analysis) were pooled again to obtain an adequate amount of material for experiments on proliferation and cytokine determination following re-stimulation with antigen. Finally, the cells were suspended in complete medium consisting of RPMI-1640 supplemented with 10% heat-inactivated fetal bovine serum (FBS), non-essential amino acids, HEPES, sodium pyruvate, and penicillin–streptomycin (Gibco). Cell counts and viability were determined using trypan blue exclusion; only preparations with viability >90% were used for downstream analyses.

All reagents used for cell culture were tissue culture grade and were purchased from SIGMA, Poznań, Poland.

### 2.5. Specific IgG and IgA Determination by Indirect ELISA

Specific IgG and IgA antibodies against PA were determined using standard indirect ELISA, as previously described [3]. Briefly, microtiter plates were coated with PA, blocked with a protein-containing buffer, and incubated with serial dilutions of serum or fecal extracts. Bound antibodies were detected using isotype-specific secondary antibodies conjugated to horseradish peroxidase (HRP) and visualized using the chromogenic substrate ABTS. The absorbance was measured at the recommended wavelength. The results were expressed as the reciprocal of the highest sample dilution, with an absorbance value 0.1 units above that of the negative control present on each ELISA plate (all reagents with PBS instead of sample).

HRP-conjugated anti-mouse IgA and IgG antibodies and PBS were purchased from SIGMA, Poznań, Poland. ABTS (Millipore, Temecula, CA, USA) was used to visualize the effect.

### 2.6. Flow Cytometry Analysis

Freshly isolated lymphocytes and those from PA-stimulated cultures were stained for surface markers using an antibody cocktail: PE anti-mouse CD8a (clone OX-8), FITC anti-mouse CD4 (clone RM4-5), and APC anti-mouse CD25 (clone PC61) (BD-Pharmingen), three colors panel. The antibodies were diluted in FACS buffer (PBS, pH 7.4, supplemented with 5% FBS), and the cell suspensions were incubated for 15 min at 4 °C in the dark. After staining, the samples were washed with FACS buffer and centrifuged at 400× *g* for 10 min at 4 °C. Cells were then fixed with 2% paraformaldehyde (PFA) and analyzed using a BD LSR Fortessa Cell Analyzer (BD Biosciences, San Jose, CA, USA) equipped with BD FACSDiva Software v 9.0 (BD Biosciences, San Jose, CA, USA). At least 50,000 events were acquired for each sample in each experiment. Data were analyzed using the FlowJo v 9.0 Software (FlowJo LLC, Ashland, OR, USA) [21]. Lymphocytes were first gated based on forward and side scatter properties to select small, low-granularity events [22], with doublets excluded using standard area/height parameters. Within the lymphocyte gate, CD4^+^ and CD8^+^ subsets were gated, and CD4^+^CD25^+^ cells were identified.

All reagents used for cytometric analyses were purchased from BD Biosciences (San Jose, CA, USA).

### 2.7. Lymphocyte Proliferation Test

A standard CFSE-based method was used for the lymphocyte proliferation test to obtain the lymphocyte proliferation index (PI) [23]. This assay allows sensitive quantification of cell division at the single-cell level and is widely used to evaluate antigen-specific T cell responses in splenocyte cultures [24,25]. Briefly, freshly isolated splenocytes were stained with CFSE and seeded (10^6^ per well) in multiwell culture plates in complete medium (RPMI 1640 supplemented with 10% heat-inactivated fetal bovine serum (FBS), 1 mM non-essential amino acids, 1 mM HEPES, 1 mM sodium pyruvate, and 10 units/mL penicillin–streptomycin solution), conditions commonly used to maintain murine splenocyte viability and function in vitro [24,25]. The cells were allowed to rest for 6 h to form a monolayer, which helped stabilize cell viability and basal activation status before stimulation [25,26], and were then stimulated with a PA solution at a concentration of 1 mg/mL. This concentration and a 96 h incubation period were selected based on our preliminary optimization experiments, which indicated that they induced a robust but non-toxic proliferative response in the splenocytes. After 96 h, the cells were harvested and analyzed using a BD LSR Fortessa Cell Analyzer (BD Biosciences, San Jose, CA, USA) equipped with BD FACSDiva Software v 9.0 (BD Biosciences, San Jose, CA, USA). The data were further processed using FlowJo v 9.0 Software (FlowJo LLC, Ashland, OR, USA) with the proliferation platform, as described in the FlowJo Proliferation Modeling User Guide.

### 2.8. Cytokine Determination

Freshly isolated splenocytes from the PP1 + PA and PA groups (Figure 1) were cultured ex vivo and stimulated with PA for 96 h. The culture supernatants were collected and stored at −80 °C until analysis [3]. IL-2, IL-4, IL-6, IL-10, IL-17A, TNF, and IFN-γ levels were measured using a Cytometric Bead Array (CBA) Mouse Th1/Th2/Th17 Cytokine Kit (560485, BD Biosciences, San Jose, CA, USA), according to the manufacturer’s instructions. Samples and standards were acquired using a BD LSR Fortessa Cell Analyzer (BD Biosciences, San Jose, CA, USA) equipped with BD FACSDiva Software v 9.0 (BD Biosciences, San Jose, CA, USA). Cytokine concentrations were calculated using FCAP Array v. 3.0 software (BD Biosciences). Assays were performed in triplicate (*n* = 3), and values below the detection limit were considered undetectable (n.d.).

### 2.9. Analysis of Short-Chain Fatty Acids (SCFAs)

The concentrations of SCFAs in the cecal contents were determined using gas chromatography, according to a standard method [27]. Briefly, the cecal samples were preserved in 0.1% trifluoroacetic acid (TFA, SIGMA, Poznań, Poland) and stored at −80 °C until analysis. After thawing, the samples were homogenized and centrifuged, and the clear supernatant was used for GC analysis. An Agilent 7890A gas chromatograph equipped with a flame ionization detector, 7683B auto-injector, and SGE BP21 capillary column was used for analysis. External standards for individual SCFAs were used to construct calibration curves and to calculate the sample concentrations.

### 2.10. Statistical Analysis

All data are presented as group means ± standard deviation (SD). Individual mice were considered experimental units (for ELISA *n* = 10, for SCFA *n* = 5), and technical replicates (e.g., triplicate for proliferation and CBA cytokine assay) were averaged before statistical analysis. Statistical analyses were performed according to the number of groups being compared. Comparisons between two groups were conducted using Welch’s corrected unpaired Student’s *t*-test, whereas comparisons among multiple groups were analyzed by one-way analysis of variance (ANOVA) followed by Tukey’s post-hoc multiple-comparison test. Differences were considered statistically significant at *p* < 0.05. Statistical analyses were performed using GraphPad Prism v. 9 (GraphPad Software, San Diego, CA, USA).

## 3. Results and Discussion

Probiotic preparations have beneficial effects on host organisms. Several studies have demonstrated the benefits of probiotics in the treatment of IBD, post-antibiotic microbiome dysbiosis, Helicobacter pylori infection, mental health, upper respiratory tract infections, infant colic, and allergic diseases [27,28,29]. The mechanisms of action of probiotics are not yet fully understood. However, they are known to reduce inflammatory processes by inhibiting the growth of pathogenic bacteria, and their metabolites affect the immune system, nervous system (for example, SCFAs that cross the blood–brain barrier), interfere with microglia, astrocytes, and neurons, and with neurotransmitters that affect immunocompetent cells in the gut [30]. Another study found that orally administered probiotics interact with intestinal epithelial cells or other immune cells, thereby increasing TLR2 receptor expression on the surface of macrophages and DCs, suggesting an important role for probiotics in the induction of the innate immune response [31]. Probiotics can influence cytokine and chemokine production, regulatory T cell activation, and splenic macrophage activation. We assumed that they could alter CD4^+^ hyper-responsiveness to food proteins. Our previous studies have shown that probiotics affect the immune cell profile and modulate the humoral response in milk protein-sensitized mice [14,27]. The present study tested the effects of probiotic supplementation on immune activity against pea albumins.

### 3.1. Probiotic Treatment Affects CD4^+^/CD8^+^ Ratio

**Figure 2:**
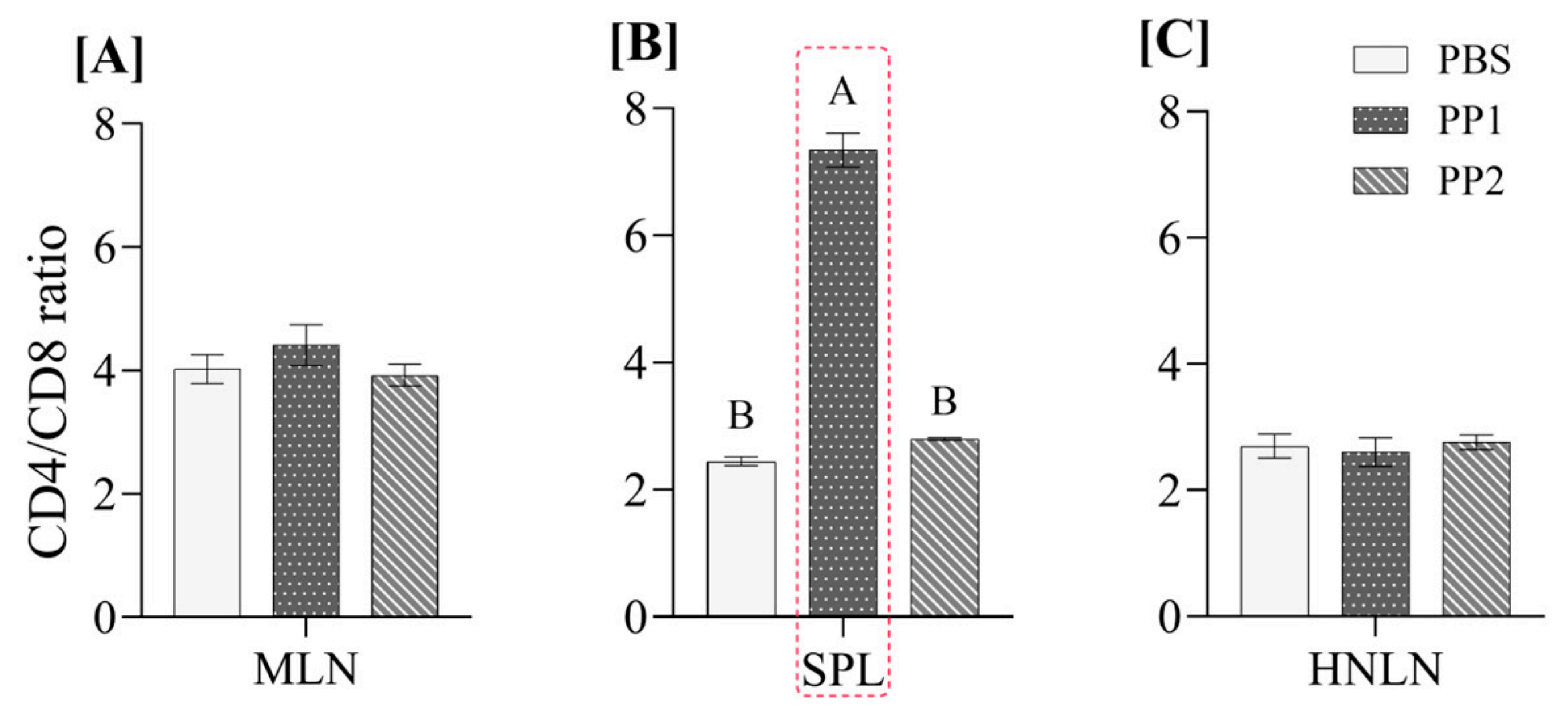
Ratio of CD4^+^/CD8^+^ lymphocytes in freshly isolated lymphoid tissues: (**A**): MLNs, (**B**): SPLs, and (**C**): HNLNs, of Balb/c mice. Control mice (PBS) were orally administered PBS, whereas the experimental groups received probiotic preparations, PP1 or PP2. The results are presented as group means ± SD (*n* = 5). Each tissue sample was analyzed individually. One-way analysis of variance (ANOVA), followed by Tukey’s post-hoc test, was used to assess statistical differences between groups. Different letters above the bars indicate statistically significant differences at *p* < 0.05.

As mentioned above, most previous studies have evaluated probiotics in the context of established inflammation or sensitization. In contrast, little is known about how probiotic exposure in otherwise healthy hosts shapes the distribution of T cell subsets and the conditions that follow immune responses to newly introduced dietary proteins, such as pea albumins. We conducted a pre-experiment to compare the effects of probiotic preparations PP1 and PP2 on the CD4^+^/CD8^+^ ratio in healthy Balb/c mice (Figure 2). The CD4^+^/CD8^+^ ratio, which reflects the balance between the two major T-cell populations, is a useful parameter for evaluating immunomodulatory interventions and disease or treatment effects in clinical settings, such as HIV infection and cancer therapy [32,33]. In our study, supplementation of naïve mice with the tested probiotics did not alter the CD4^+^/CD8^+^ ratio in the MLNs, the main inductive site for immune responses to food allergens [34], or in the peripheral HNLNs (Figure 2). In contrast, we found a marked effect in the spleen: the CD4^+^/CD8^+^ ratio in splenocytes from PP1-treated mice was almost three times higher than that in the PBS and PP2 groups (*p* < 0.05; Figure 2). These findings indicate that the multi-strain preparation PP1 modulated the T cells profile in the spleen, a key effector organ, more efficiently than the single-strain preparation PP2. The spleen is critical for the development of both innate and adaptive immunity and is a central site where naïve lymphocytes are activated by circulating antigens, leading to the generation of specific antibodies and effector CD4^+^ and CD8^+^ cells [35,36].

Metabolites produced by probiotic strains are important mediators of T cell modulation [37]. Bacterial metabolites circulate in the blood to the spleen, where they can influence resident lymphocytes, which then recirculate to other lymphoid tissues. Multi-strain formulations, such as PP1, may generate a broader spectrum and higher levels of metabolites than single-strain formulations. However, these mechanisms were not directly assessed in this study and should be regarded as plausible explanations rather than established pathways for the observed effects. We observed that PP1 had a distinct effect, whereas PP2 did not. Therefore, PP1, but not PP2, was selected for subsequent experiments.

**Figure 3:**
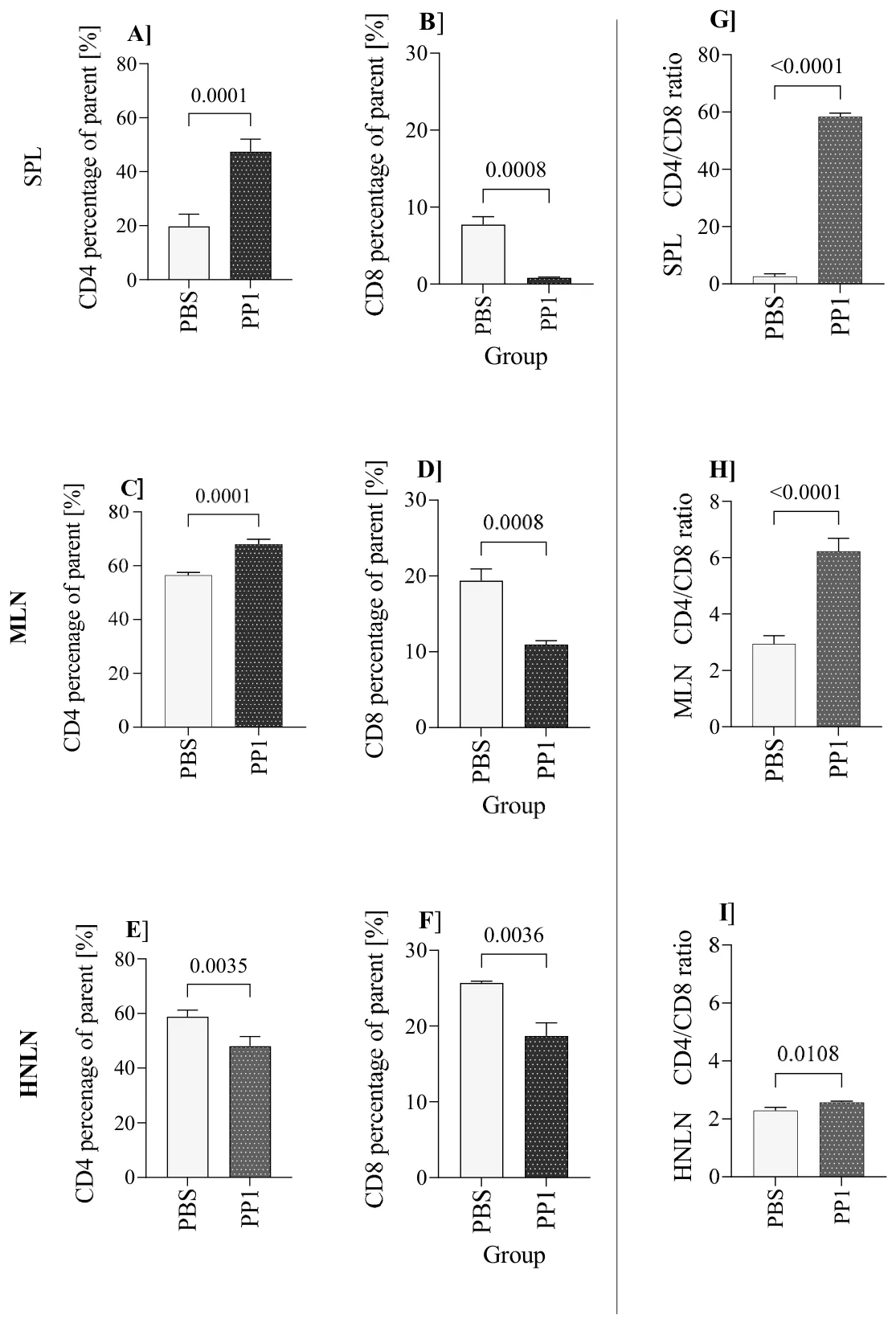
Percentages of CD4^+^ and CD8^+^ lymphocyte populations in stimulated ex vivo lymphocytes from SPLs (**A**,**B**), MLNs (**C**,**D**), and HNLNs (**E**,**F**) of Balb/c mice and the resulting CD4/CD8 ratio (**G**–**I**). Balb/c mice were orally administered PBS (control) or a probiotic preparation (PP1). Isolated cells were stimulated ex vivo with pea albumin (PA). The results are presented as group means ± SD (*n* = 5). Differences in the percentages of lymphocyte populations following stimulation were assessed using an unpaired *t*-test with Welch’s correction, comparing the probiotic-treated group with the PBS control group. Statistical significance was set at *p* < 0.05.

To pre-evaluate how PP1-induced changes in vivo might influence T cell responses to pea albumins, lymphocytes from the SPLs, MLNs, and HNLNs of PP1- and PBS-treated mice were stimulated ex vivo with PA. We then monitored the activity of CD4^+^, CD4^+^CD25^+^, and CD8^+^ populations in the tissues that are critical for adaptive immunity (Figure 3). In PP1-treated mice, we observed an increase in CD4^+^ cells and a decrease in CD8^+^ cells in all tissues tested, except for the HNLNs, where CD4^+^ cells were reduced compared to the PBS group (*p* < 0.05; Figure 3). As expected for naïve mice without prior antigen exposure, there were no significant differences in the CD4^+^CD25^+^ population between the groups confirming the absence of antigen-specific regulatory T cell expansion at this stage. Nevertheless, the CD4^+^/CD8^+^ ratio was significantly increased in all tissues of PP1-treated mice compared to that of the PBS group (*p* < 0.05; Figure 3).

### 3.2. Humoral Response

**Figure 4:**
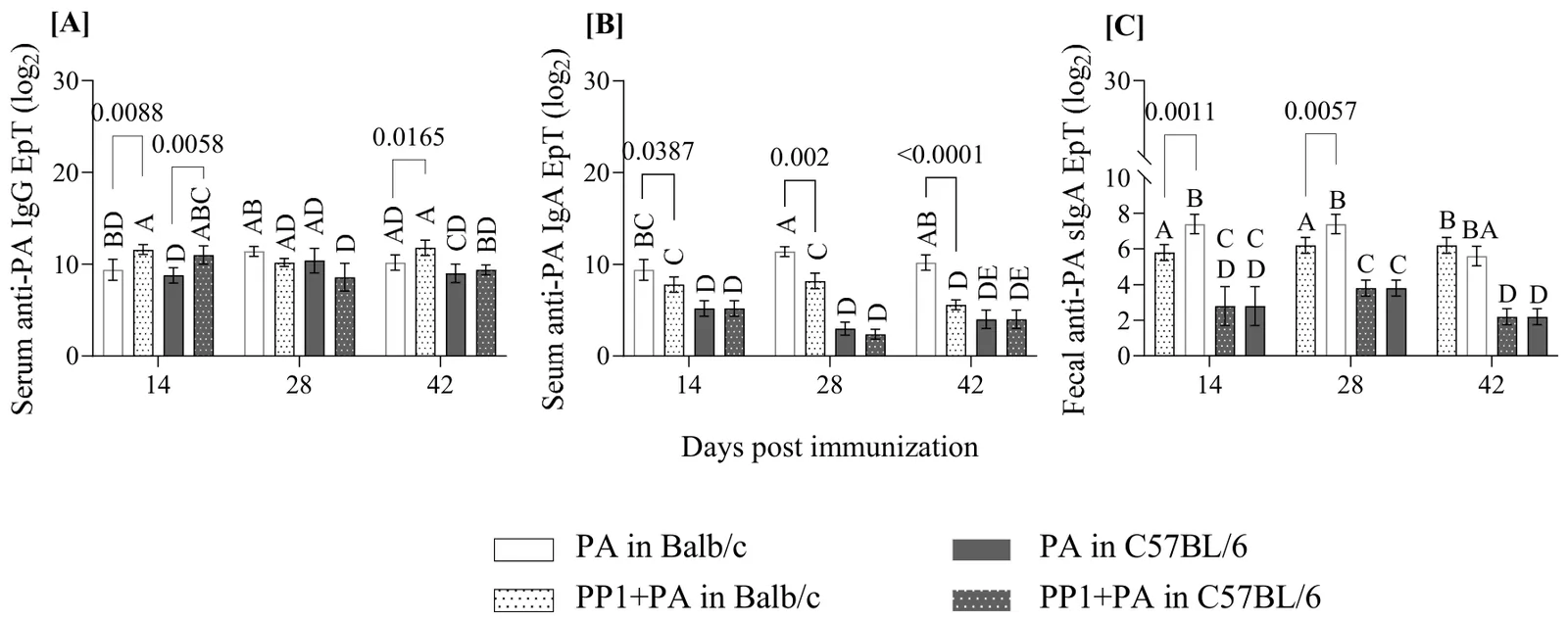
Humoral response of mice to pea albumin on days 14, 28, and 42 after antigen administration. Balb/c and C57BL/6 mice received PP1 probiotic preparation with pea albumin (PA) via oral gavage, while the control groups received PA alone. Specific ani-PA serum IgG (**A**), IgA (**B**) and fecal secreted IgA (**C**) are presented as mean ± SD (*n* = 10). The day-point effect of PP1 on anti-PA antibody levels was assessed using a *t*-test with Welch’s correction (*p* < 0.05). The effects of mouse strain and treatments between time points were analyzed using one-way ANOVA followed by Tukey’s post-hoc test, with *p* < 0.05 considered statistically significant. Different capital letters indicate statistically significant differences.

Based on the results of the preliminary experiment, a subsequent study was conducted in which Balb/c and C57BL/6 mice were administered PBS, PA, or a PP1 + PA mixture (Figure 1). Starting on day 14 following the first antigen administration, the humoral response to PA was monitored in all experimental groups (Figure 4). Consistent with our previous findings [3], C57BL/6 mice exhibited lower levels of PA-specific immunoglobulins than Balb/c mice (*p* < 0.05; Figure 4). C57BL/6 mice displayed an early increase in serum anti-PA IgG, followed by relatively stable antibody titers, whereas Balb/c mice showed a more pronounced and sustained response following PP1 administration (Figure 4A). The anti-PA serum IgG titer in Balb/c mice was significantly elevated at the first checkpoint on day 14 and remained increased until day 42 (Figure 4A, *p* = 0.0165). Likewise, significant increases in fecal IgA levels were observed at nearly all analyzed time points following PP1 treatment in Balb/c mice (Figure 4C). Serum sIgA was significantly lower compare to PA group (Figure 4B), but higher (*p* < 0.05) then C57BL/5 mice. In contrast, PP1 administration did not significantly alter serum or fecal antigen-specific IgA levels in C57BL/6 mice (Figure 4B, C).

The strain-dependent differences observed in our study are consistent with previous reports demonstrating distinct microbiota compositions and IgA-associated immune phenotypes in Balb/c and C57BL/6 mice [38,39]. According to the literature, Balb/c mice exhibit greater microbiota diversity, higher IgA abundance, and a more diverse IgA repertoire than C57BL/6 mice, which may contribute to enhanced mucosal and antigen-specific antibody responses [39]. Moreover, unlike C57BL/6 mice, germ-free Balb/c mice produce polyreactive IgA that facilitates the generation of antigen-specific IgA responses and influences microbiota diversification [39]. Consistent with these findings, probiotic-mediated immune effects have been shown to vary between mouse strains according to their baseline microbiota composition and host background [38]. Therefore, although microbiota composition was not assessed in the present study, pre-existing strain-specific differences in microbiota and IgA responsiveness may have contributed to the enhanced antibody responses observed in Balb/c mice compared to C57BL/6 mice [38,39].

### 3.3. Cellular Immune Response

**Figure 5:**
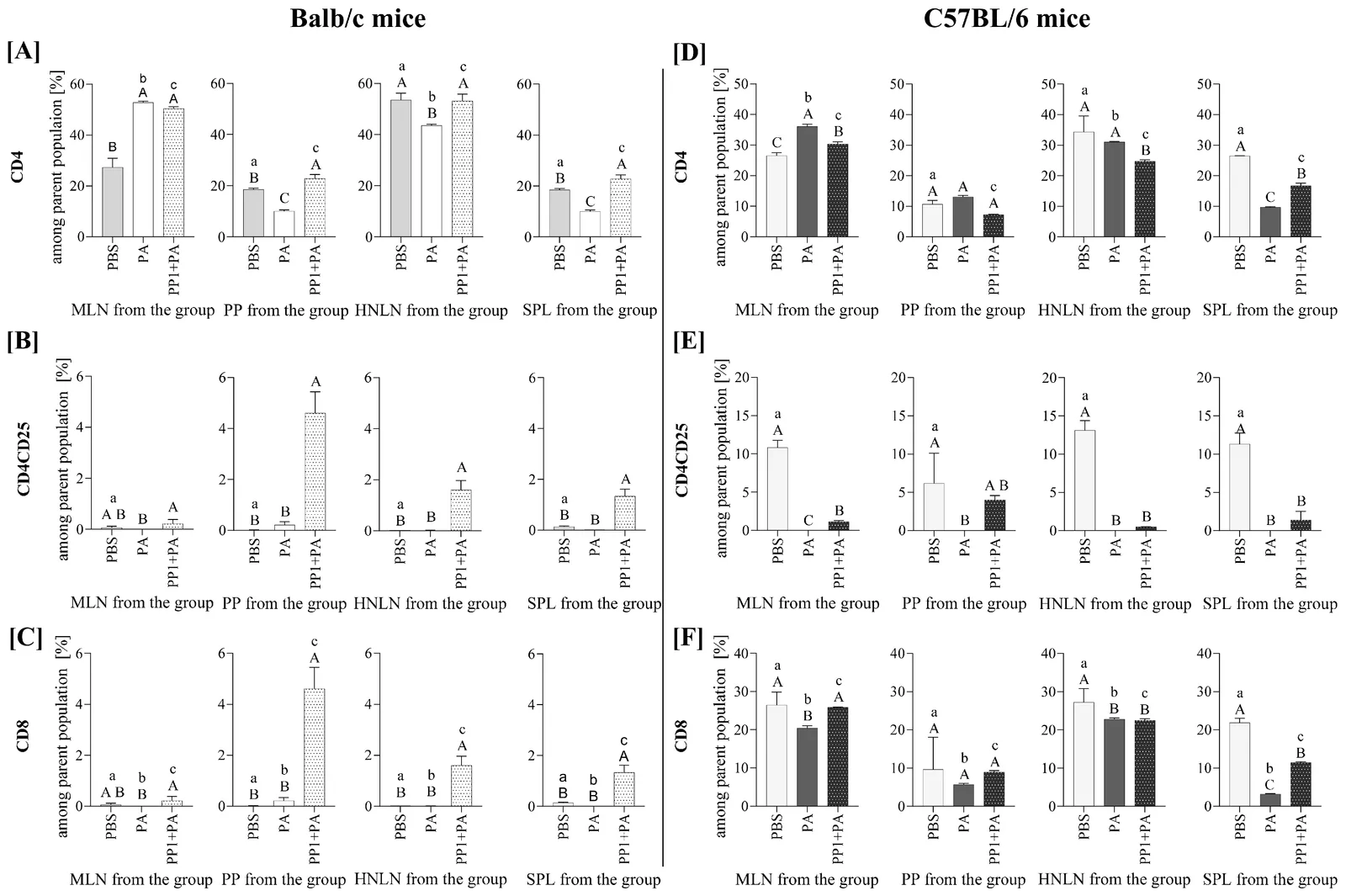
CD4^+^, CD4^+^CD25^+^, and CD8^+^ lymphocyte populations in the lymphoid tissues (MLNs, PPs, HNLNs, and SPLs) of Balb/c (**A**–**C**) and C57BL/6 (**D**–**F**) from experimental groups: PBS, control mice treated with PBS; PA, mice treated with pea albumin; PP1 + PA, mice treated with probiotic PP1 and pea albumin. Results are presented as the mean of the group ± SD (the tissues from each experimental group were pooled (3, 3, and 4), resulting in *n* = 3). One-way analysis of variance (ANOVA) with Tukey’s test was used to adjust for statistical differences between groups. Bars with different capital letters are significantly different at *p* < 0.05. Comparisons were made separately for each tissue and each population. Differences between mice strains (Balb/c vs. C57BL/6) receiving the same treatment in the same tissues were adjusted using the *t*-test with Welch’s correction. Statistical significance was set at *p* < 0.05. Significant differences between strains are indicated with the same small letter up to the bar: ‘a’ for PBS, ’b’ for PA, and ‘c’ for PP1 + PA treatment.

We found that the effect of PP1 probiotic on modulating the humoral immune response was closely related to the mouse strain and specific IgA levels (Figure 4). However, it is important to assess the activity of probiotics and their effects on immune responses at the cellular level. We phenotyped CD4^+^, CD4^+^CD25^+^, and CD8^+^ T-cell subsets in the MLNs, PPs, HNLNs, and SPLs of Balb/c and C57BL/6 mice treated with PBS, PA, or a PP1 + PA mixture (Figure 5).

Administration of probiotic PP1 with pea albumins, and PA alone almost doubled the CD4^+^ cell population in MLNs of Balb/c mice compared to PBS group (*p* < 0.05). It proofs MLNs are the induction site of immune response to food allergen. The significantly higher percentage were observed also in PPs and SPLs of Balb/c mice (*p* < 0.05 vs. PBS and PA groups; Figure 5A). Simultaneously, there was a significant increase in the populations of CD4^+^CD25^+^ and CD8^+^ cells in PPs and HNLNs compared to the PBS and PA groups (*p* < 0.05; Figure 5B, C). Peyer’s patches (PPs) are the first site of immune response induction and are crucial for establishing tolerance to administered antigens or allergenic sensitization to food components. Exposure of the germinal centers of the PPs to gut antigens and microbiota, as well as probiotic preparations, makes them the site of induction and modulation of various immune cells and immunoglobulin class A [40]. In contrast to Balb/c mice, C57BL/6 mice exhibited only limited alterations in tested lymphocyte populations following treatment (Figure 5D–F). No significant changes were observed in the PPs, with the exception of a reduction in CD4^+^CD25^+^ regulatory T cells after PA treatment (*p* < 0.05). In the MLNs, both PA and PP1 + PA treatment significantly increased the percentage of CD4^+^ cells compared with the PBS group (*p* < 0.05). In HNLN and SPL the PP1 + PA and PA group showed a significantly lower percentage of CD4^+^ compared to PBS groups (*p* < 0.05). Notably, the percentage of CD4^+^CD25^+^ cells was significantly decreased in all tissues examined following both PA and PP1 + PA administration. Similarly, CD8^+^ cell frequencies were either reduced or remained comparable to those observed in the PBS group (*p* < 0.05). Collectively, these findings indicate that the immunomodulatory effects of PA and PP1 + PA in C57BL/6 mice were modest and tissue-specific, being primarily characterized by a reduction in regulatory T-cell populations rather than by enhanced T-cell responses. The Balb/c mice showed an increase in the CD4^+^ population in the PPs, HNLNs, and SPLs as a result of PP1 treatment compared to the PA group (*p* < 0.05; Figure 5A). As shown above, PP1 probiotic treatment affected specific IgA and sIgA titers in Balb/c mice but not in C57BL/6 mice (Figure 4). The population of CD8^+^ cells in C57BL/6 mice was generally higher than that in Balb/c mice (*p* < 0.05; Figure 5). Human studies have shown that IgA induces the regulated expression of its receptors on dendritic cells and various inflammatory responses in CD8^+^ cells [41]. We found that the percentage of CD8^+^ lymphocytes significantly increased in the MLNs and SPLs of C57BL/6 mice following probiotic intervention compared to the group administered PA only (*p* < 0.05; Figure 5F). However, no effect on the humoral response was observed, which may be due to the genotypically more stable immune system of these mice and the resident microbiome, making the effects of the administered probiotics less pronounced [42,43]. The slight increase in CD8^+^ cells in Balb/c mice treated with PP1 + PA (*p* < 0.05) suggests that probiotic supplementation may influence T-cell response during allergen exposure. However, because the gut microbiota was not analyzed in the present study, the contribution of microbiota-derived mechanisms to this effect remains unclear. The absence of this impact after PA treatment alone or in C57BL/6 mice (Figure 5F), indicate that host genetic background and the interaction of probiotic and allergen exposure may be important determinates of immunomodulatory effect. Given the crucial role of CD8^+^ cells in immune vigilance and protection against infection and cancer [44] those findings warrant further investigation.

During the study we assessed the proliferative activity of splenocytes isolated from the experimental mice following ex vivo re-exposure to the antigen (Supplementary Figure S1). Although splenocytes from each experimental group presented different proliferation index values after PA stimulation, 1.35 ± 0.14, 1.63 ± 0.06 and 1.38 ± 0.17 respectively for PBS, PA and PP1 + PA treated group, no significant differences were observed among the Balb/c groups. In the C57BL/6 strain, only splenocytes from the PP1 + PA group displayed a significantly higher proliferative response compared with the PBS control group (*p* = 0.0365). Furthermore, no strain-dependent differences in proliferative activity were detected.

### 3.4. Cytokine Secretion in Response to Antigen Re-Exposure

The proliferation assay is crucial for assessing lymphocyte activation and understanding the immunological landscape in response to different antigens. However, differences in cytokine profiles between groups provide further insights into their functional capacity for immunogenic cell activity. The extent to which ex vivo stimulation reflects the in vivo immune response is largely unknown. Jansen et al. [45] found that in the case of monocytes, ex vivo and in vitro responses did not reflect immune responses to induced human endotoxemia in a cohort of volunteers. However, in the case of food antigens, the profile of cytokines secreted ex vivo demonstrates the strength of protein antigenicity and the activity of immune cells against the antigens. This study highlights the potential implications of developing protein modifications for targeted therapies and dietary interventions to modulate allergic responses. Cytokines released by immunocompetent cells (e.g., lymphocytes and macrophages) are soluble messengers of the immune system that regulate the activity of immunogenic cells and develop immune responses. We determined the cytokine profiles released by primary cultures of lymphocytes isolated from the effector tissue spleen (SPL) of PA- or PA + PP1-treated Balb/c mice in response to ex vivo re-exposure to pea albumin to verify the differences in immune cell activity after PP1 treatment (Table 1). There were no differences in the proliferation index (PI) between the strains in the ex vivo test (Figure S1); therefore, we excluded C57BL/6 lymphocytes from cytokine testing.

Isolated spleen cell suspensions reflect the majority of immune cells present in the spleen: CD4^+^ lymphocytes, dendritic cells (DCs), and macrophages, which collectively contribute to cytokine production during immune response. Dietary antigens enter the spleen via the bloodstream, where they are presented to lymphocytes by dendritic cells and macrophages. Following antigen recognition, some of the antigen-activated lymphocytes remain in the spleen, whereas others enter the bloodstream and migrate to specialized lymphoid tissues, such as the HNLN. Probiotic intervention in vivo reduced the immune response to PA, as evidenced by the lower cytokine secretion following ex vivo stimulation of splenocytes with PA. The strong cytokine response observed following ex vivo PA stimulation indicates that splenocytes were primed and retained antigen-specific reactivity. However, in mice treated with probiotics, this response was attenuated, resulting in reduced production of Th1/Th2 cytokines (Table 1).

A dramatic decrease in the concentration of pro-inflammatory cytokines was observed, particularly TNF-α, whose concentration decreased from 609.27 ± 9.90 in the PA group to 217.22 ± 5.17 pg/mL in the PP1 + PA group (*p* < 0.0001). The concentration of the cytokine IL-4, which is associated with the pathogenesis of allergic diseases, fell by almost threefold from 59.11 ± 2.05 pg/mL in the splenocyte culture from the PA group to 17.03 ± 0.98 pg/mL in the case of cells from the group treated with the probiotic (*p* < 0.0001). Collectively, these findings indicate that PP1 attenuates PA-induced immune activation by suppressing both Th1- and Th2- associated cytokine responses. Simultaneously, an increase in CD4^+^ and CD4^+^CD25^+^ lymphocyte levels was observed in the SPL cultures of PP1 + PA group (Figure 5A,B). Significant amounts of TNF-α and IL-6 determined in restimulated cultures can secreted by antigen-activated macrophages [46]. However, in murine splenocytes, the macrophage content is approximately 3.5% of the total cells [47]. Therefore, it can be generalized that the detected cytokines are mainly secreted by CD4^+^ and CD8^+^ cells. Furthermore, an active response to antigen re-exposure in peripheral lymphoid tissues, such as HNLN (Table S1), demonstrates efficient homing activated lymphocyte in the peripheral lymph nodes. Ex vivo, the HNLN released several cytokines responsible for active T cell differentiation into Th2, Th1 and Th 17, respectively IL-4 and IL-10, IL-2, IL-6, TNF-α and IFN-γ and IL-17. As for the pleiotropic cytokines IL-4 and IL-10, their concentrations varied among tissues and mainly decreased (Table S1), suggesting effective silencing of Th2 cells in the inductive tissues. In contrast, high levels of TNF-*α* and IL-6 in PP (Table S2) indicate that the probiotic preparation effectively activated lymphocytes and macrophages in induction tissue [48]. The effects of probiotics require detailed studies of the communication pathways between immunocompetent cells and probiotic metabolites at the molecular scale.

### 3.5. Short-Chain Fatty Acids

**Figure 6:**
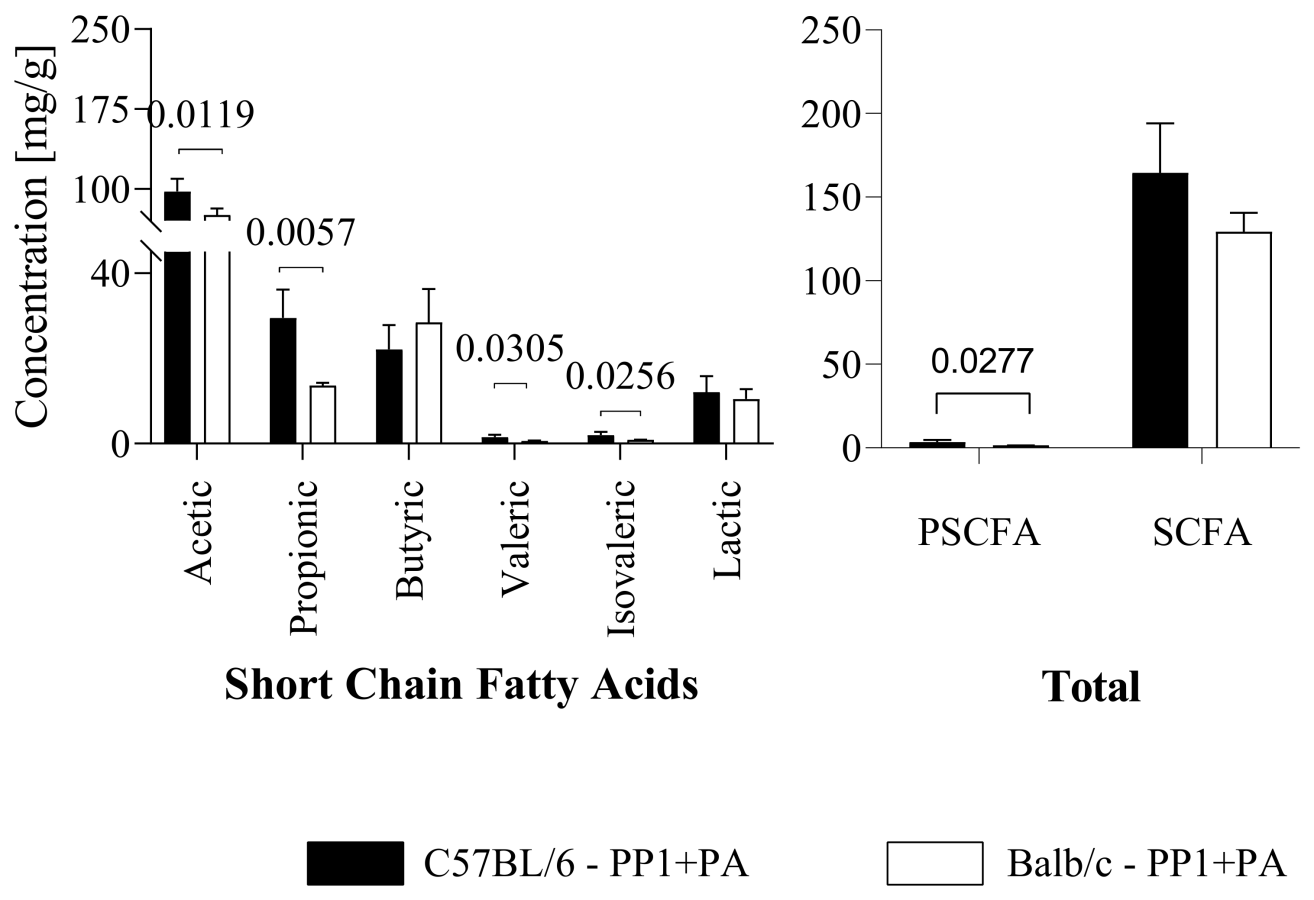
SCFAs profiles in the cecal contents of Balb/c and C57BL/6 mice treated with PP1 probiotic preparation and pea albumins (PP1 + PA group). PSCFAs—the sum of the putrefactive fatty acids (valeric and isovaleric). SCFAs—the sum of short-chain fatty acids (acetic, propionic, butyric, valeric, isovaleric, and lactic acid). Data are presented as mean ± SD (*n* = 5 per group). Statistical differences between mouse strains were evaluated using a *t*-test with Welch’s correction. Statistical significance was defined as *p* < 0.05.

SCFAs signal through G protein-coupled receptors GPR43 (FFAR2) and additionally propionate and butyrate through GPR41/FFAR3, with butyrate also acting via GPR109A; these SCFA-receptor interactions are linked to neutrophil chemotaxis, T cell differentiation, activation, and subsequent cytokine production [49]. Independent of receptor signaling, butyrate and propionate act intracellularly as histone deacetylase inhibitors, producing anti-inflammatory effects on intestinal epithelial cells, macrophages, and dendritic cells, and have been implicated in promoting the development of Treg cells possibly other T cell subsets [40,50], which is very important in the development of allergy sites [50,51]. Butyrate conditions dendritic cells toward a tolerogenic phenotype, and priming naïve CD4^+^ T cells toward IL-10-producing type 1 regulatory T cells (Tr1) through combined HDAC inhibition and GPR109A signaling [52]. With respect to secreted immune factors, SCFAs directly influence B cell biology, for instance, by enhancing oxidative phosphorylation, glycolysis, and fatty acid synthesis in B cells and promoting antibody production [53,54]. We quantified the SCFA concentrations in the cecal contents of Balb/c and C57BL/6 mice as a first-line readout to determine whether probiotic supplementation altered the metabolic activity of the gut microbiota (Figure 6). The profiles of major SCFAs differed between strains after PP1 at the level of propionic and two putrefactive acids (propionic 30 ± 9 vs. 12 ± 3 mg/mL, *p* = 0.0057; valeric *p* = 0.0305, isovaleric *p* = 0.0256 for C57BL/6 and Balb/c. mice respectively). These results suggest that PP1 treatment was associated with strain-specific changes. SCFAs, such as acetate, propionate, and butyrate, support gut health and modulate immune responses by influencing T cell differentiation and cytokine production [48,55]. Butyrate serves as a primary energy source for colonocytes, promotes intestinal barrier integrity, and can induce effector and regulatory T cells through histone deacetylase inhibition and mTOR-S6K pathway regulation [48]. Putrefactive SCFAs, valeric and isovaleric, showed significantly higher levels in C57BL/6 mice compared to Balb/c. A cohort study (110 children) concluded that higher concentrations of fecal valeric acid may be characteristic of a protective gut microbiota and/or actively contribute to allergy protection [56]. Although our study was not designed to dissect the causal links between SCFA shifts and immune outcomes, the combination of altered SCFA profiles and strain-dependent differences in humoral and cellular responses suggests that microbiota-derived metabolites may contribute to the distinct immunomodulatory effects observed in Balb/c and C57BL/6 mice.

### 3.6. Limitations of the Study

This study had several limitations that should be considered when interpreting the results. First, only two inbred strains, Balb/c and C57BL/6, were used in this study. They differ in genetic background, baseline Th polarization (Th2-biased and Th1-biased, respectively) [19,57], and likely in baseline gut microbiota composition. This design allowed us to assess whether the findings were consistent across different immune backgrounds; however, strain-dependent differences, such as those in IgA, cannot be attributed to a single factor, and the Th1/Th2 interpretation remains hypothetical. C57BL/6 mice are reported to be more susceptible to anaphylactic shock. Anyway, no signs of behavioral abnormalities were observed in any of the strains in the present study. That suggesting that Th1/Th2 labels alone do not predict the response to PA in this model. Additional strains (e.g., C3H/HeJ), 16S rRNA or metagenomic profiling, cohousing or microbiota-transfer experiments, and broader profiling of other lymph nodes and intestinal lamina propria lymphocytes are needed to establish the mechanisms of strain-dependent responses.

Second, gut microbiota composition was not analyzed. The SCFAs were measured only in PP1 + PA treated groups, as an indirect indicator of microbial metabolic activity. Therefore, we cannot determine whether the probiotic altered microbiota composition or whether microbiota differences underlie the strain-specific IgA responses.

Third, the study was conducted exclusively in female mice, which limits the generalizability of the results. Sex-dependent differences in the release of alarmins, IgE class switching, mast cell reactivity, and microbiota composition have been increasingly recognized [58,59,60]. Including both sexes in future experiments would allow for a more comprehensive assessment of the influence of sex on the dynamics of sensitization and resulting allergic pathology.

Fourth, all animals were fed a high-quality, sterilized, standardized, commercial TPF diet (1314 and 1324 TPF; Altromin). This approach facilitated methodological transparency and study replication by using a single fixed-formula commercial diet. However, these natural-ingredient diets based on cereal and soybean components do not provide the same level of compositional control and consistency as purified diets [61]. Therefore, potential variability inherent to natural-ingredient diets should be considered when interpreting the findings. Incorporating varied dietary conditions into future studies may help elucidate how nutritional composition modulates the severity of sensitization, intestinal barrier integrity, and microbiota resilience.

Fifth, sampling tissues with low cell yields, such as Peyer’s patches, imposes practical limitations on the scope of immunophenotyping achievable with the current sample sizes. Peyer’s patches provide key information on mucosal immune activation; however, their limited cell content reduces the reliability of further analyses when the number of samples is small. Future research projects will include larger experimental groups to ensure an adequate number of cells for high-resolution profiling of mucosal lymphoid compartments.

Sixth, the proliferation index (PI) in this study was determined for the total splenocyte population rather than for individual cell subsets. Consequently, although specific cell populations may have expanded at different rates, these changes could have been masked when analyzing the bulk splenocyte mixture, resulting in an overall unchanged PI.

## 4. Conclusions

In summary, the probiotic preparation PP1 tested in this study showed immunomodulatory capacities in mouse strains in a strain-dependent manner. Balb/c mice responded to pea albumins; therefore, they appear to be a good model for studying food protein immunoreactivity. The administration of the multi-strain probiotic PP1 to Balb/c mice challenged with pea albumins altered their humoral and cellular responses to the antigens. A significant increase in CD4^+^ and CD8^+^ cell population activity was observed at the sites of induction of the immune response to food antigens. Fluctuations in specific IgG titers, increased IgA titers, and reduced cytokine secretion by lymphocytes ex vivo in response to antigen re-exposure indicate the significant potential of probiotics to achieve a balanced immune response in the host. Given the known role of SCFAs in modulating cellular and humoral responses, the observed strain-dependent changes in SCFAs levels in mice suggest host microbiome-dependent modulation.

The studies conducted explored several promising avenues that may influence the personalization of the immune response through the controlled delivery of probiotics and antigens, both in combination and separately. They also raise multiple questions regarding the synergistic effect of multi-strain probiotic preparations or treatment regimens (schedule and dose of probiotic or antigen), which were not part of those studies. Inducer lymphoid tissues (MLN, PP) and effector tissues (SPL) show potential as key sites for the induction and effective storage of antigen-specific cells. However, although the mouse model remains a valuable tool in preclinical research, limitations regarding reproducibility in the human context still exist. First, the tissue-specific migration of lymphoid cells to Peyer’s patches and mesenteric lymph nodes—the primary induction sites analyzed here—cannot be directly assessed in humans. This necessitates the identification of noninvasive, peripherally accessible biomarkers (e.g., circulating cytokine profiles, fecal SCFAs, or immunoglobulin signatures) that reliably reflect the mucosal immune response. Humanized mouse models, which are constantly being refined and developed, serve as an additional bridge between mouse and human models. Second, studies have observed strain-dependent variability in both the response and SCFA levels among mice. This raises the question of whether the response to probiotics is determined by a permissive genetic background. This corresponds to the heterogeneity of allergic predisposition and immune reactivity in the human population. This variability justifies the need for further research using genetically diverse or humanized mouse models to better elucidate the underlying mechanisms of this variability. Future studies should prioritize the validation of systemic surrogate biomarkers in parallel with expanded genetic and microbiome-humanized models to establish more direct correlations between probiotic intervention, the immune response, and gut microbiota activity in a manner that is translatable to the human population.

## Figures and Tables

**Table 1:** Cytokine levels secreted by splenocytes isolated from Balb/c mice administered pea albumins (PAs) or the PP1 probiotic preparation combined with PA via intragastric gavage. Ex vivo splenocyte cultures were stimulated with PA. Data are presented as mean ± SD (*n* = 3). Statistical differences between the treatments were evaluated using a *t*-test with Welch’s correction. Statistical significance was set at *p* < 0.05.

Cytokine	Stimulation Agent	Cytokine Concentration [pg/mL]		*p* Value	Different
		Experimental Groups			
		PP1 + PA	PA		
TNF-α	Medium	6.25 ± 1.91	47.47 ± 3.12	0.0001	Yes
	PA	217.22 ± 5.17	609.27 ± 9.90	<0.0001	Yes
IFN-γ	Medium	0.28 ± 0.27	3.96 ± 0.29	0.0018	Yes
	PA	88.30 ± 0.33	542.46 ± 4.74	<0.0001	Yes
IL-2	Medium	1.93 ± 0.69	6.88 ± 1.20	0.0033	Yes
	PA	928.15± 31.03	4998.19 ± 108	<0.0001	Yes
IL-6	Medium	6.13 ± 0.72	21.26 ± 1.15	<0.0001	Yes
	PA	74.54 ± 1.82	151.81 ± 2.57	<0.0001	Yes
IL-17	Medium	n.d.	0.3 ± 0.026	-	-
	PA	15.0 ± 0.53	108.92 ± 2.41	<0.0001	Yes
IL-10	Medium	6.84 ± 0.26	8.84 ± 0.08	0.0245	Yes
	PA	35.45 ± 3.17	270.98 ± 18.01	0.0015	Yes
IL-4	Medium	2.93 ± 0.77	2.54 ± 0.87	0.4729	No
	PA	17.03 ± 0.98	59.11 ± 2.05	<0.0001	Yes

## Data Availability

The original contributions presented in this study are included in the article/Supplementary Material. Further inquiries can be directed to the corresponding authors.

## Supplementary Materials

The following supporting information can be downloaded at: https://www.mdpi.com/article/10.3390/nu18203403/s1, Figure S1. Proliferation Index of splenocytes isolated from Balb/c and C57BL/6 mice after experimental treatment stimulated with pea albumins (PAs). Groups: PBS, control mice treated with PBS; PA, mice treated with pea albumins; PP1 + PA, mice treated with probiotic PP1 and pea albumins. Results are presented as the mean of the group ± SD (*n* = 5). One-way analysis of variance (ANOVA) followed by Tukey’s post-hoc test was used to assess differences among treatment groups within each mouse strain separately (Balb/c and C57BL/6). Comparisons between strains within the same treatment group were performed using an unpaired *t*-test with Welch’s correction. A *p*-value < 0.05 was considered statistically significant. Table S1. Cytokine levels secreted by HNLN-derived lymphocytes isolated from Balb/c mice administered pea albumin (PA) or PP1 probiotic preparation combined with PA via intragastric gavage. Ex vivo lymphocyte cultures were stimulated with PA. Data are presented as mean ± SD (*n* = 3). Statistical significance between groups was evaluated using the *t*-test with Welch’s correction; *p* < 0.05 was considered statistically significant. Table S2. Cytokine levels secreted by PP-derived lymphocytes isolated from Balb/c mice administered pea albumin (PA) or PP1 probiotic preparation combined with PA via intragastric gavage. Ex vivo lymphocyte cultures were stimulated with PA. Data are presented as mean ± SD (*n* = 3). Statistical significance was evaluated using the *t*-test with Welch’s correction; *p* < 0.05 was considered statistically significant.

## Abbreviations

The following abbreviations are used in this manuscript:

CMA	Cow Milk Allergy
PA	Pea albumin
PP 1,2	Probiotic preparation 1,2
CD	Cluster of differentiation
GALT	Gut-associated lymphoid tissue
SCFA	Short-Chain Fatty Acid
IL-	Interleukin
TNF-α	Tumor necrosis factor α
IFN-γ	Interferon γ
DC	Dendritic cell
Th	T helper cell
SPL	Spleen
MLN	Mesenteric lymph node
HNLN	Head and neck lymph node
PP	Peyer’s patches
PBS	Phosphate-buffered saline
ELISA	Enzyme-linked immunosorbent assay
