# Supplementary material for: Impact of Probiotic Intervention on Lymphocyte Activity in Mice Challenged with Pea Albumins (Pisum sativum L.)
Source: Nutrients. 2026 Oct 17;18(20):3403. doi: 10.3390/nu18203403 (PMC13708179; doi:10.3390/nu18203403)
Supplement: Supplementary file 1 [file nutrients-18-03403-s001.zip › 2026_Nutrients_Zlotkowska_ImpactProbioticsCytokiny_TableS1_2.pdf]

Impact of Probiotic Intervention on Lymphocyte Activity in Mice Challenged with Pea Albumins (*Pisum sativum* L.)

Justyna Chudzik-Kozłowska [1,*], Ewa Wasilewska [1], Anita Mikołajczyk [2] and Dagmara Złotkowska [1,*]

[1] InLife Institute of Animal Reproduction and Food Research, Polish Academy of Sciences, 10-683 Olsztyn, Poland; e.wasilewska@pan.olsztyn.pl
[2] Department of Psychology and Sociology of Health and Public Health, Collegium Medicum, University of Warmia and Mazury in Olsztyn,, 10-082 Olsztyn, Poland; a.mikolajczyk@uwm.edu.pl
* Correspondence: j.chudzik-kozlowska@wp.pl (J.C.-K.); d.zlotkowska@pan.olsztyn.pl (D.Z.)

Table S1. Cytokine levels secreted by HNLN-derived lymphocytes isolated from Balb/c mice administered pea albumin (PA) or PP1 probiotic preparation combined with PA via intragastric gavage. Ex vivo lymphocyte cultures were stimulated with PA. Data are presented as mean ± SD (*n=3*). Statis-tical significance between groups was evaluated using the t-test with Welch's correction; $p < 0.05$ was considered statistically significant.

Cytokines	Stimulation agent	Cytokine concentration [pg/mL] Experimental groups		p value	Different
		**PP1+PA**	**PA**		
TNF - α	Medium	2.91 ± 0.50	10.83 ± 1.68	0.0098	Yes
	PA	405.28 ± 1.82	55.28 ± 0.15	<0,0001	Yes
IFN - γ	Medium	n.d.	0.68 ± 0.09		
	PA	92.63 ± 2.60	0.39 ± .01	0.0003	Yes
IL - 2	Medium	1.92 ± 0.09	n.d.		
	PA	112.63± 1.78	21.18 ± 0.65	<0.0001	Yes
IL - 6	Medium	0.81 ± 0.02	1.91 ± 1.28	0.2719	No
	PA	34.2 ± 1.41	4.86 ± 1.46	<0,0001	Yes
IL - 17	Medium	n.d.	0.3 ± 0.026	-	-
	PA	15.0 ± 0.53	108.92 ± 2.41	<0.0001	Yes
IL - 10	Medium	2.42 ± 0.23	n.d.		
	PA	4.43 ± 0.15	270.98 ± 18.01	0.0035	Yes
IL-4	Medium	2.28 ± 1.21	2.22 ± 0.71	0.9453	No
	PA	11.44 ± 0.14	2.25 ± 0.15	<0.0001	Yes

Table S2. Cytokine levels secreted by PP-derived lymphocytes isolated from Balb/c mice administered pea albumin (PA) or PP1 probiotic preparation combined with PA via intragastric gavage. Ex vivo lymphocyte cultures were stimulated with PA. Data are presented as mean ± SD (*n=3*). Statistical significance was evaluated using the t-test with Welch's correction; $p < 0.05$ was considered statistically significant

**Cytokines**	**Stimultion agent**	**Cytokine concentration [pg/mL]**		p value	**Different**
		**Experimental groups**			
		**PP1+PA**	**PA**		
TNF - α	Medium	6.28 ± 1.35	4.05 ± 1.09	0.093	Yes
	PA	29.06 ± 3.58	10.37 ± 3.64	0.0032	Yes
IFN - γ	Medium	n.d.	n.d.	-	
	PA	n.d.	n.d.	-	
IL - 2	Medium	1.77 ± 0.06	3.04 ± 0.32		
	PA	928.15± 31.03	4998.19 ± 108	<0.0001	Yes
IL - 6	Medium	7.46 ± 0.46	1.21 ± 0.44	-	
	PA	25.81 ± 1.43	n.d.	-	
IL - 17	Medium	n.d.	n.d.	-	
	PA	n.d.	n.d.	-	
IL - 10	Medium	n.d.	5.78 ± 0.42	-	
	PA	n.d.	2.81 ± 0.23	-	
IL-4	Medium	1.89 ± 0.10	1.85 ± 0.13	0.6996	No
	PA	0.95 ± 0.25	1.73 ± 0.18	0.0146	Yes
