# Supplementary material for: Impact of Probiotic Intervention on Lymphocyte Activity in Mice Challenged with Pea Albumins (Pisum sativum L.)
Source: Nutrients. 2026 Oct 17;18(20):3403. doi: 10.3390/nu18203403 (PMC13708179; doi:10.3390/nu18203403)
Supplement: Supplementary file 1 [file nutrients-18-03403-s001.zip › 2026_Nutrients_Zlotkowska_PI_Fig.S1.pdf]

Impact of Probiotic Intervention on Lymphocyte Activity in Mice Challenged with Pea Albumins (*Pisum sativum* L.)

Justyna Chudzik-Kozłowska [1,*], Ewa Wasilewska [1], Anita Mikołajczyk [2] and Dagmara Złotkowska [1,*]

[1] InLife Institute of Animal Reproduction and Food Research, Polish Academy of Sciences, 10-683 Olsztyn, Poland; e.wasilewska@pan.olsztyn.pl
[2] Department of Psychology and Sociology of Health and Public Health, Collegium Medicum, University of Warmia and Mazury in Olsztyn,, 10-082 Olsztyn, Poland;
a.mikolajczyk@uwm.edu.pl
* Correspondence: j.chudzik-kozlowska@wp.pl (J.C.-K.); d.zlotkowska@pan.olsztyn.pl (D.Z.)

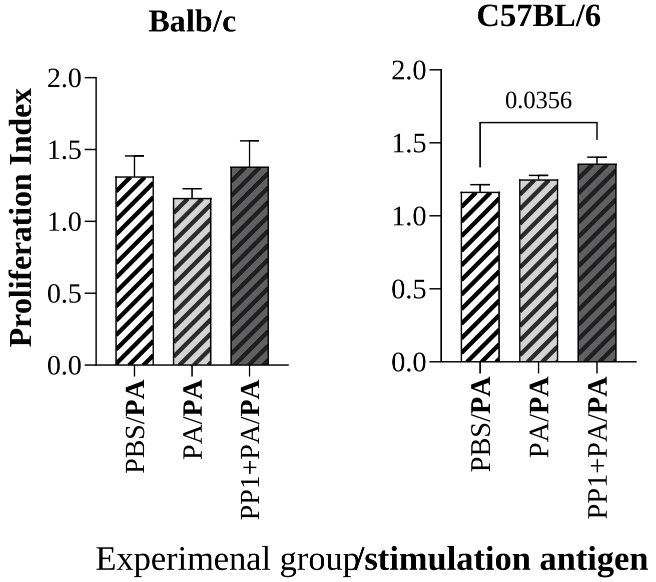

Figure S1. Proliferation Index of splenocytes isolated from Balb/c and C57BL/6 mice after experimental treatment stimulated with pea albumins (PA). Groups: PBS, control mice treated with PBS; PA, mice treated with pea albumins; PP1+PA, mice treated with probiotic PP1 and pea albumins. Results are presented as the mean of the group ± SD (*n= 5*). One-way analysis of variance (ANOVA) followed by Tukey's post hoc test was used to assess differences among treatment groups within each mouse strain separately (BALB/c and C57BL/6). Comparisons between strains within the same treatment group were performed using an unpaired t-test with Welch's correction. A p-value < 0.05 was considered statistically significant.
